# Expression of Cadherin-17 Promotes Metastasis in a Highly Bone Marrow Metastatic Murine Breast Cancer Model

**DOI:** 10.1155/2017/8494286

**Published:** 2017-01-19

**Authors:** Tomoko Okada, Atsushi Kurabayashi, Nobuyoshi Akimitsu, Mutsuo Furihata

**Affiliations:** ^1^Biomedical Research Institute, National Institute of Advanced Industrial Science and Technology (AIST), 1-1-1 Higashi, Tsukuba, Ibaraki 305-8566, Japan; ^2^Department of Pathology, Kochi Medical School, Nankoku, Kochi 783-8505, Japan; ^3^Radioisotope Center, The University of Tokyo, Bunkyo-ku, Tokyo 113-0032, Japan

## Abstract

We previously established 4T1E/M3 highly bone marrow metastatic mouse breast cancer cells through* in vivo* selection of 4T1 cells. But while the incidence of bone marrow metastasis of 4T1E/M3 cells was high (~80%) when injected intravenously to mice, it was rather low (~20%) when injected subcutaneously. Therefore, using 4T1E/M3 cells, we carried out further* in vitro* and* in vivo* selection steps to establish FP10SC2 cells, which show a very high incidence of metastasis to lungs (100%) and spines (85%) after subcutaneous injection into mice. qRT-PCR and western bolt analysis revealed that cadherin-17 gene and protein expression were higher in FP10SC2 cells than in parental 4T1E/M3 cells. In addition, immunostaining revealed the presence of cadherin-17 at sites of bone marrow and lung metastasis after subcutaneous injection of FP10SC2 cells into mice. Suppressing cadherin-17 expression in FP10SC2 cells using RNAi dramatically decreased the cells' anchorage-independent growth and migration* in vitro* and their metastasis to lung and bone marrow* in vivo*. These findings suggest that cadherin-17 plays a crucial role in mediating breast cancer metastasis to bone marrow.

## 1. Introduction

Bone is a main target of breast cancer metastasis such that nearly 80% of patients with advanced breast cancer suffer from bone metastasis [1, 2]. And because once bone metastasis has occurred, the quality of life of the affected breast cancer patient is seriously diminished, new approaches to preventing and reducing bone metastasis are urgently required. We previously established the 4T1E/M3 highly bone marrow metastatic breast cancer cell line [3] and showed that the chemokine CCL2/MCP-1 negatively regulates the cells' growth, migration, and metastasis [4], while BMP7 signaling increases the metastatic potential of these cells [5]. However, although the metastatic potential of 4T1E/M3 cells is high (about 80%) when injected intravenously into mice, it is only 20% to 30% when injected subcutaneously [3]. In the present study, we established more highly metastatic breast cancer cell line, FP10SC2, which shows 85% to 100% metastasis to spine when injected subcutaneously into mice.

Cadherin-17, also known as liver-intestine cadherin or human peptide transporter-1, is a structurally unique member of the cadherin superfamilies [6, 7]. Whereas classical cadherins have five cadherin repeats, cadherin-17 contains seven cadherin repeats and is closely associated with cadherin-16 in the seven cadherin domain subfamily. In addition, cadherin-17 has only about 20 amino acids in its cytoplasmic domain, while classical cadherins contain a highly conserved cytoplasmic domain of 150–160 amino acids. Cadherin-17 is expressed in mice and humans almost exclusively in epithelial cells of both embryonic and adult small intestine and colon, fetal liver, and B lymphocytes [8], where it plays an important role during embryonic gastrointestinal development and also functions as a peptide transporter. In cancer, cadherin-17 expression reportedly correlates with clinical association with tumor metastasis and advanced tumor stages in hepatocellular carcinoma [9], knockdown of cadherin-17 inhibited cell proliferation, adhesion, migration, and invasion in gastric cancer [10], and an anti-cadherin-17 antibody suppresses subcutaneous hepatocellular carcinoma growth and lung metastasis [11]. In the present study, we found that expression of cadherin-17 in our highly metastatic FP10SC2 cells was higher than that in the parental 4T1E/M3 cells and that suppressing cadherin-17 expression reduced the metastatic potential of FP10SC2 cells. These results suggest that the expression of cadherin-17 promotes breast cancer metastasis to bone marrow.

## 2. Materials and Methods 

### 2.1. Animals

Seven- to eight-week-old female BALB/c mice were purchased from Japan Clea (Tokyo, Japan).

### 2.2. Establishment of the FP10SC1 and FP10SC2 Cell Lines

The 4T1E/M3 highly bone metastatic mouse breast cancer cell line was established as described previously [3]. Briefly, 4T1 murine breast cancer cells were initially transfected with a plasmid harboring the neomycin resistance gene and selected in medium (RPMI 1640 supplemented with 2 mM L-glutamine, 1.5 g/L sodium bicarbonate, 4.5 g/L glucose, 10 mM HEPES, 1.0 mM sodium pyruvate, and 10% fetal bovine serum: GIBCO, Invitrogen, Carlsbad, CA, USA) containing 120 *μ*g/mL G418 (Invitrogen). The selected cells were injected into the tail vein of BALB/c mice (1 × 10^6^ cells/mouse), and 12 days later bone marrow cells were recovered from the femurs and tibias by flushing. These cells were cultured in the medium containing 120 *μ*g/mL G418 and intravenously injected into a new set of mice, and this cycle was repeated three times to establish the 4T1E/M3 cells. When 4T1E/M3 cells were intravenously injected into mice, the incidence of metastasis to the spine was about 80%, but it was only 20% when the cells were injected subcutaneously [3].

To establish cells with greater metastatic potential after subcutaneous injection, 4T1E/M3 cells (2 × 10^5^/mL, 200 *μ*L/well) were seeded onto polycarbonate membranes (8 *μ*m pores) within transwell chambers (Krabo, Osaka, Japan) in 24-well plastic tissue culture plates. After incubation for 2 days, the cells that migrated through the pores in the membrane were harvested and seeded onto a new membrane. After repeating this process 10 times, the cells were subcutaneously injected into BALB/c mice (1 × 10^6^ cells/mouse), and 25 days later mice were sacrificed and bone marrow cells were collected from the spines by flushing the bones using a 25G needle and syringe (Terumo Co. Ltd., Tokyo, Japan). The collected cells were cultured in medium containing 120 *μ*g/mL G418, and FP10SC1 cells were established. FP10SC1 cells (1 × 10^6^ cells/mouse) were then subcutaneously injected into a new set of mice, and this cycle was repeated to establish FP10SC2 cells.

### 2.3. Cell Proliferation Assay

Cells (1 × 10^4^/mL, 200 *μ*L/well) were seeded into 96-well plates and cultured for 0 to 4 days, and 10 *μ*L of Cell Counting Kit-8 reagent (Dojin Laboratories, Tokyo, Japan) was added to each well. After the additional 4 h incubation at 37°C under 5% CO_2_, the absorbance at 450 nm was measured using a microplate reader (Model 680, Bio-Rad Laboratories, Inc., Hercules, CA).

### 2.4. Anchorage-Independent Proliferation Assay

Anchorage-independent colony formation in soft agar was assayed as described previously [5]. First, culture medium containing 0.5% agarose (Difco noble agar: BD Diagnostic Systems, Sparks, MD, USA) was added to 6 cm dishes (5 mL/dish). Thereafter, cells (1 × 10^4^/mL, 2 mL/dish) suspended in culture medium containing 0.3% agarose (Difco noble agar) were layered onto the 0.5% agarose in the dishes. The cells were then cultured for 12 days at 37°C under 5% CO_2_, after which the total numbers of colonies per dish were counted under a microscope.

### 2.5. *In Vitro* Invasion Assay

The* in vitro* invasion assay system used was described previously [12]. Briefly, cells (2 × 10^5^/mL, 200 *μ*L/well) were seeded onto polycarbonate membranes (8 *μ*m pores) in transwell chambers (Krabo) and placed in 24-well plates. For transendothelial invasion assays, a bone marrow derived endothelial cell (BMEC) monolayer [13] was formed on fibronectin-coated membranes, and the cells (2 × 10^5^/mL, 200 *μ*L/well) were seeded onto the monolayer. After incubation for 18 h, the cells that migrated through the pores to the underside of the membrane were counted under a microscope.

### 2.6. *In Vivo* Tumor Experiments

Cells (1 × 10^6^/mouse) were subcutaneously injected into the backs or mammary fad pad of BALB/c mice. Twenty-five days later, the mice were sacrificed and their lungs and spines were collected and subjected to histological examination. Each experiment was repeated by three times. All animal studies were reviewed and approved by the Institutional Animal Care and Use Committee of National Institute of Advanced Industrial Science and Technology (AIST).

### 2.7. Histology

Lungs and spines from mice were fixed in 10% neutral buffered formalin (Wako Pure Chemical Industries, Ltd., Osaka, Japan), processed, embedded in paraffin, sectioned at 5-6 *μ*m, stained with H&E, and examined and photographed under a microscope. The spines were decalcified using decalcifying solution A (Plank Rychlo Method, Wako Pure Chemical Industries) following the manufacturer's protocol before embedding in paraffin. The percent metastatic area per tissue in the histological sections was calculated using Image J software. For immunohistochemical analysis, specimens were stained using rabbit anti-cadherin-17 IgG (sc-25628; Santa Cruz Biotech Inc., Santa Cruz, CA, USA) and HRP-conjugated goat anti-rabbit IgG (R&D).

### 2.8. RNA Isolation and Quantitative RT-PCR

Total RNA isolation and cDNA synthesis were performed as described previously [3]. The reverse transcription protocol entailed incubation at 25°C for 10 min, 55°C for 30 min, 85°C for 5 min, and 0°C for 5 min. Specific forward and reverse primers were designed using Primer 3 software (Genetyx Co., Tokyo, Japan) and synthesized by Fasmac Co. Ltd. (Kanagawa, Japan). The primer sequences (5′-3′) used were as follows: for GAPDH (glyceraldehyde-3-phosphate dehydrogenase, product size, 209 bp), CCCCTTCATTGACCTCAACTAC (forward) and TGGTGGTGAAGACACCAGTAGA (reverse); for cadherin-17 (product size, 180 bp), ACTGAAGTAGGTGGGTCCTCT (forward) and CCGAAGTGACTGCTGGTCAT (reverse). The quantitative RT-PCR (qRT-PCR) reactions were set up according to the Light Cycler manual (Roche Diagnostics GmbH, Mannheim, Germany) using a Light Cycler Fast Start DNA Master SYBR Green I Kit (Roche Diagnostics). The PCR protocol entailed denaturation at 95°C for 10 min, 45 cycles of denaturation at 95°C for 10 s, annealing at 59°C for 10 s, and extension at 72°C for 11 s, and a final cooling step at 40°C for 30 s. For each reaction, the crossing point (defined as the cycle number at which the noise band intersected the fluorescence curves) was determined using the “Second Derivative Maximum Method” in the Light Cycler software (ver. 3.52, Roche Diagnostics). Relative mRNA levels were calculated using the software with a standard curve constructed using various concentrations of a 1 : 1 mixture of the RT product and then normalized to GAPDH mRNA.

### 2.9. RNA Interference

For vector-based RNAi analysis, we synthesized miRNA oligo DNA (CTGTACAAGTAAGCTAAGCACTTCGTGGCCGTCGATCGTTTAAAGGGAGGTAGTGAGTCGACCAGTGGATCCTGGAGGCTTGCTGAAGGCTGTATGCTGAACACAGGCACTTCATTCACAGTTTTGGCCACTGACTGACTGTGAATGGTGCCTGTGTTCAGGACACAAGGCCTGTTACTAGCACTCACATGGAACAAATGGCCCAGATCTGGCCGCACTCGAGATATCTAGACCCAGCTTTCTTGTACAAAGTGGTTG) using Cadherin17 BLOCK-iT™ miR RNAi Select (Invitrogen), Mmi 506198_top_Cadherin17, and Mmi 506198_bot_Cadherin17 (Invitrogen) to generate knockdown clones numbers 1 and 2. The sequence was inserted into an expression vector to obtain pcDNA6.2-GW/EmGFP-miR-mCDH17, the sequence of which was verified by Life Technologies Inc. (Tokyo, Japan). The plasmid pcDNA6.2-GW/EmGFP-miR-neg (Invitrogen), which carried a scrambled sequence and could not target any known vertebrate gene, was served as a control nontargeting vector. To knock down the targeted genes, FP10SC2 cells were transfected with the vector using FuGene HD (Roche Diagnostics) following the manufacturer's protocol. Cells were then cultured in medium containing 2 *μ*g/mL blasticidin (Wako, Tokyo, Japan) to yield stable control/FP10SC2 and Cadherin-17(−)/FP10SC2 clones numbers 1 and 2 after limiting dilution.

### 2.10. Western Blotting

Cells were seeded into 10 cm dishes and treated with Complete Lysis buffer (Roche Diagnostics) following the manufacturer's protocol, after which the protein obtained was measured using a BCA Protein Assay Kit (Pierce. Rochford IL, USA). The proteins were then resolved by SDS-PAGE and transferred to i-Blot Gel Transfer Stacks PVDF membranes (Invitrogen) using an i-Blot Dry Blotting system (Invitrogen). After blocking the membranes with 5% skim milk, they were incubated overnight at room temperature with anti-cadherin-17 (1/200, rabbit polyclonal IgG, sc-25628; Santa Cruz Biotech Inc., Santa Cruz, CA, USA) or anti-*β*-actin (1/2000, rabbit polyclonal IgG, sc-7210; Santa Cruz Biotech Inc.) diluted in Can Get signal immunoreaction Enhancer Solution 1 (Toyobo, Tokyo, Japan). The membranes were then washed three times with TBS-T and incubated for 1 h at room temperature with HRP-conjugated anti-rabbit IgG (1/5000, GE Healthcare) diluted in Can Get signal immunoreaction Enhancer Solution 2 (Toyobo, Tokyo, Japan). Finally, the membranes were treated with ECL Prime detection reagent (GE Healthcare) according to manufacturer's protocol, and the chemiluminescence was detected using Chemi Doc XRS (Bio-Rad Laboratories Inc.).

## 3. Results

### 3.1. Newly Established FP10SC1 and FP10SC2 Cells Showed Enhanced Spontaneous Bone Marrow Metastasis

**Figure 1:**
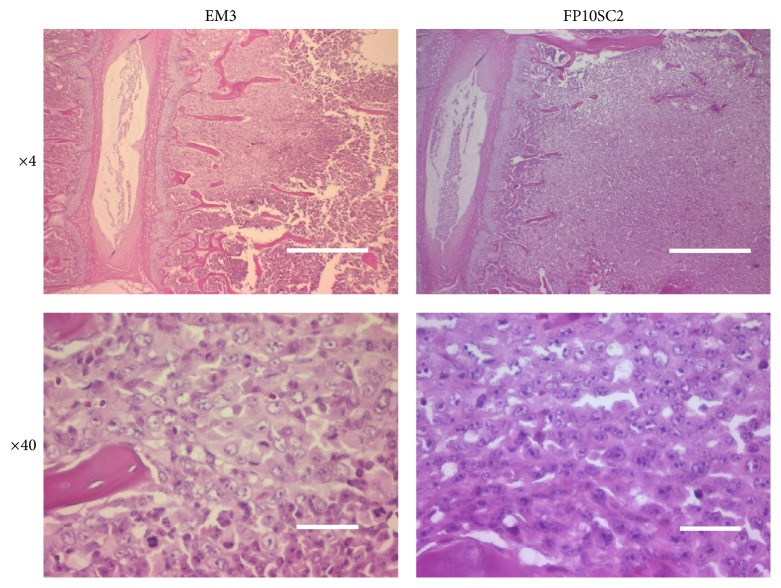
Histological examination of the spines of mice subcutaneously injected with 4T1E/M3 or FP10SC2 cells. Shown are representative histological sections of spines. Cancer metastasis in spine samples collected 25 days after injection of cells (1 × 10^6^ cells/mouse) was detected using H&E staining. Images are shown at objective lens magnifications of 4x and 40x; the bars indicate 500 and 25 *μ*m, respectively.

We established FP10SC1 cells from parental 4T1E/M3 cells through 10* in vitro* selection steps and 1* in vivo* selection step. From the FP10SC1 cells, we then established FP10SC2 cells through another* in vivo* selection steps. Histological sections of lungs and spines collected from mice subcutaneously injected with FP10SC2 or 4T1E/M3 cells after 25 days revealed the presence of metastasis in all lungs from 4T1E/M3- and FP10SC2-injected mice. In addition, metastasis was detected in the spines of 20% to 33% (2/10 or 2/6) of 4T1E/M3-injected mice and 85% (11/13) of FP10SC2-injected mice (Table 1). It means that the metastatic potential was strongly augmented in FP10SC2 cells. The photomicrographs in Figure 1 show metastasis in spines from mice subcutaneously injected with 4T1E/M3 and FP10SC2 cells.

### 3.2. Proliferation Rates of FP10SC1 and FP10SC2 Was Not Changed

**Figure 2:**
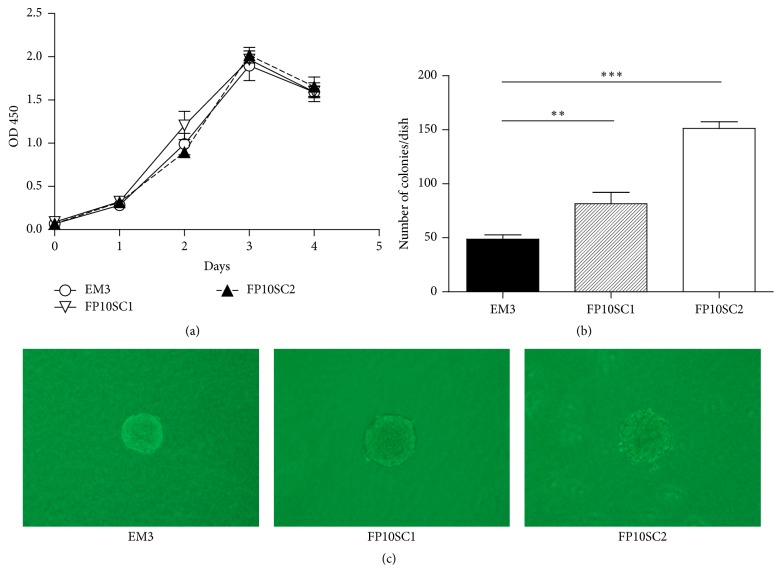
Growth curves and anchorage-independent growth of 4T1E/M3, FP10SC1, and FP10SC2 cells. (a) Growth curves for the 4T1E/M3, FP10SC1 and FP10SC2 cells measured in MTT assays. Data are means ± SD. (b) Anchorage-independent growth of 4T1E/M3, FP10SC1 and FP10SC2 cells measured in colony formation assays. Cells (1 × 10^4^/dish, 3 dishes/cell type) were cultured for 12 days in medium containing 0.3% agarose layered on 0.5% agarose. Data are means ± SD; ^*∗∗*^*p* < 0.01; ^*∗∗∗*^*p* < 0.001. (c) Representative images of colonies.

To investigate the mechanism underlying the enhanced metastatic activity of FP10SC1 and FP10SC2, we carried out modified MTT assays to examine their* in vitro* proliferation rates. As shown in Figure 2(a), there was no difference in the proliferation rates of 4T1E/M3, FP10SC1, and FP10SC2 cells.

### 3.3. Anchorage-Independent Growth of FP10SC2 Cells Was Markedly Accelerated

Anchorage-independent proliferation is a hallmark of tumor cell malignancy. We therefore assessed the anchorage-independent proliferation of FP10SC1, FP10SC2, and 4T1E/M3 cells in soft agar colony formation assays. Twelve days after plating, colonies were evident in medium containing 0.3% agar. FP10SC1 cells formed nearly twice as many colonies as did 4T1E/M3 cells, while FP10SC2 cells formed more than three times as many colonies as 4T1E/M3 cells (Figure 2(b)). The diameters of individual colonies did not differ among the three cell types (Figure 2(c)).

### 3.4. Migration of FP10SC1 and FP10SC2 Cells Was Significantly Enhanced

**Figure 3:**
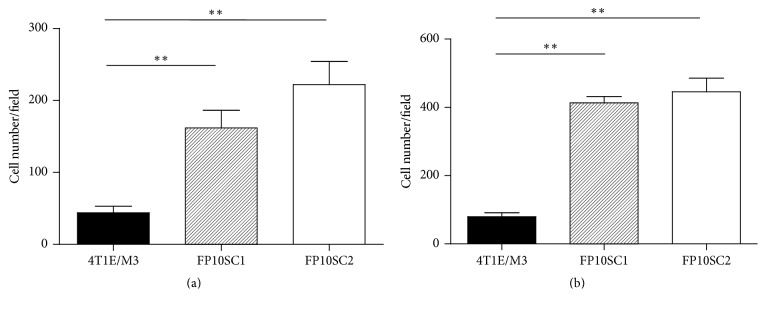
Migration of 4T1E/M3, FP10SC1, and FP10SC2 cells. (a) Cells (4 × 10^4^ cells/well) were seeded onto BMEC monolayers grown on polycarbonate porous membranes, and the numbers of cells migrating through the pores were counted after 18 h. (b) Cells (4 × 10^4^ cells/well) were seeded directly onto polycarbonate porous membranes, and the numbers of the cells migrating through the pores were counted after 18 h. In both panels, data are means ± SD; ^*∗∗*^*p* < 0.01.

The ability to migrate is another important feature of metastatic tumor cells. We therefore assessed the migration activity of FP10SC1 and FP10SC2 cells using transwell chambers in which the insert bottom was a polycarbonate membrane with 8 *μ*m pores. To measure transendothelial invasion activity, we grew a BMEC monolayer [14] on fibronectin-coated polycarbonate membranes. After seeding FP10SC1, FP10SC2, or 4T1E/M3 cells onto polycarbonate membranes, with or without BMEC monolayers, and incubating the cells for 18 h, the numbers of cells that migrated through the membrane pores were counted. As shown in Figures 3(a) and 3(b), the migration activity of FP10SC1 and FP10SC2 cells was more than 3-4 times higher than that of 4T1E/M3 cells.

### 3.5. Cadherin-17 Expression Was Dramatically Increased in FP10SC2 Cells

**Figure 4:**
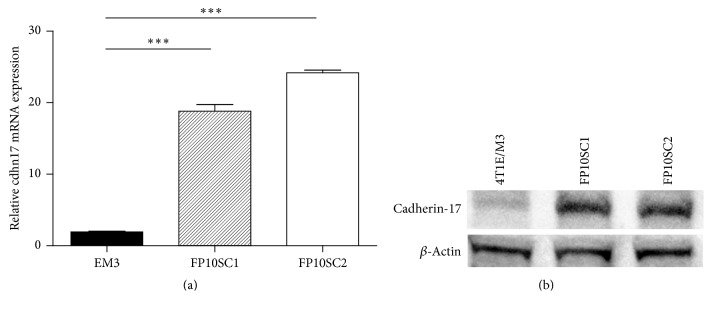
Expression of cadherin-17 in 4T1E/M3, FP10SC1, and FP10SC2 cells. (a) Quantitative real-time RT-PCR (qRT-PCR) analysis of cadherin-17 expression in 4T1E/M3, FP10SC1 and FP10SC2 cells. Levels of cadherin-17 mRNA are shown relative to that of GAPDH mRNA. Values are means ± SD; ^*∗∗∗*^*p* < 0.001. (b) Western blot analysis of cadherin-17 in lysates of 4T1E/M3, FP10SC1, and FP10SC2 cells.

Cadherin-17 is reportedly overexpressed in liver, stomach, intestinal, and pancreatic cancers, and the increased expression is associated with the occurrence of metastasis [9]. We therefore used qRT-PCR analysis to compare the levels of cadherin-17 gene expression in FP10SC1 and FP10SC2 cells with that in the parental 4T1E/M3 cells. We found that, as compared to 4T1E/M3 cells, expression of cadherin-17 mRNA was markedly (about 10 times) increased in both FP10SC1 and FP10SC2 cells (Figure 4(a)). In addition, as shown in Figure 4(b), cadherin-17 protein expression in FP10SC1 and SC2 was augmented measured by western blot analysis. We also examined the gene expression of some molecules including cadherin and integrin family and found that the cadherin-17 gene expression was significantly augmented among other adhesion molecules in FP10SC1 and FP10SC2.

**Figure 5:**
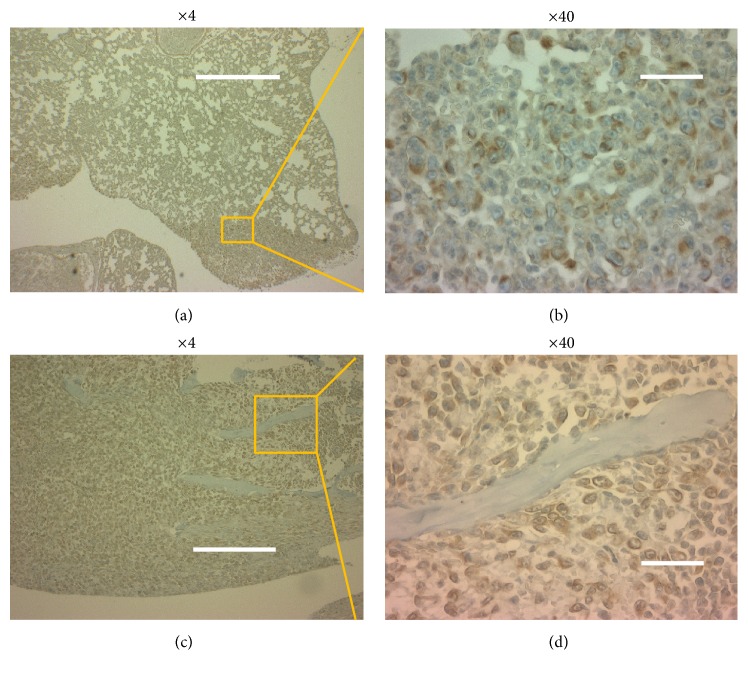
Immunohistochemical examination of the spines and lungs of BALB/c mice subcutaneously injected with FP10SC2 cells. (a)–(d) Immunostaining for cadherin-17 in spine (a, b) and lung (c, d) samples collected 28 days after subcutaneous injection of FP10SC2 cells into the backs of BALB/c mice (1 × 10^6^ cells/mouse). Objective lens magnification, (a) 4x; (b) 40x; (c) 4x; (d) 40x; the bars indicate 500 (a, c) and 25 *μ*m (b, d), respectively.

Moreover, immunohistochemical analysis of sections of lungs and spines collected 28 days after subcutaneous injection of FP10SC2 cells into mice revealed the clear presence of cadherin-17-positive metastatic cancer cells in both the lungs (Figures 5(a) and 5(b)) and spines (Figures 5(c) and 5(d)). This suggests that cadherin-17 is highly expressed* in vivo* at sites of metastasis.

### 3.6. Knocking down Cadherin-17 Gene Expression Did Not Affect* In Vitro* Proliferation

**Figure 6:**
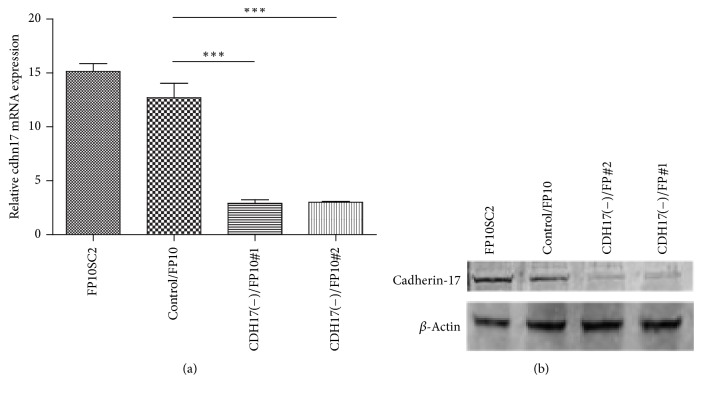
Establishment of cadherin-17 knockdown FP10SC2 clones and expression of cadherin-17. (a) qRT-PCR analysis of cadherin-17 expression. Levels of the indicated mRNAs in FP10SC2, control/FP10SC2, CDH17(−)/FP10SC2 clone #1, and CDH17(−)/FP10SC2 clone #2 are shown relative to the level of GAPDH mRNA. Values are means ± SD; ^*∗∗∗*^*p* < 0.001. (b) Western blot analysis of cadherin-17 in lysates of FP10SC2 cells, control/FP10SC2, CDH17(−)/FP10SC2 clone #1, and CDH17(−)/FP10SC2 clone #2.

**Figure 7:**
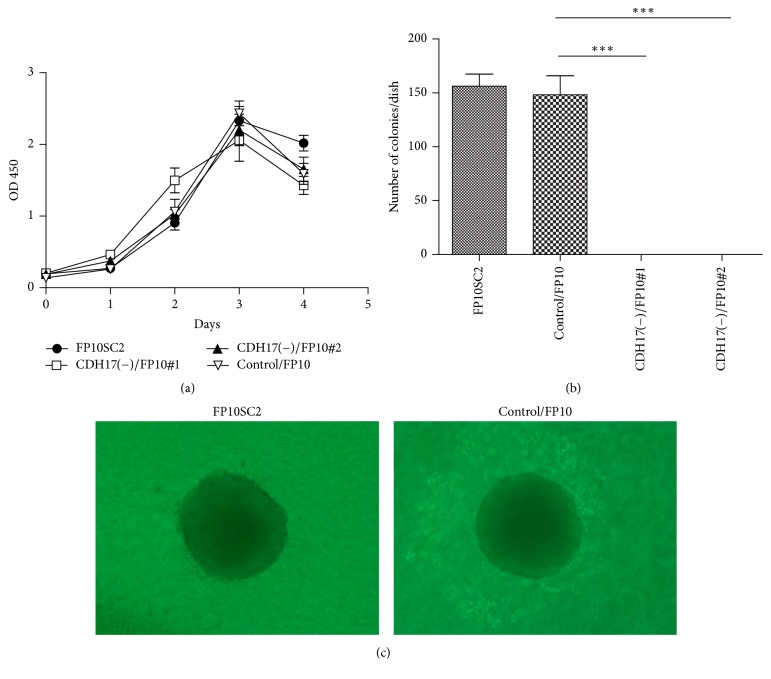
Growth curves and anchorage-independent growth of FP10SC2 cells, control/FP10SC2, CDH17(−)/FP10SC2 clone #1, and CDH17(−)/FP10SC2 clone #2. (a) Growth curves for the indicated cells measured in MTT assays. Data are means ± SD. (b) Anchorage-independent growth of the indicated cells measured in colony formation assays. Cells (1 × 10^4^/dish, 3 dishes/cell type) were cultured for 12 days in medium containing 0.3% agarose layered on 0.5% agarose. Data are means ± SD; ^*∗∗∗*^*p* < 0.001. (c) Representative images of colonies.

To assess the effects of the augmented cadherin-17 expression on the behavior of FP10SC2 cells, we established two stable transfectant clones, cadherin-17(−)/FP10SC2#1 and #2, in which cadherin-17 was knocked down by miR RNAi. Using qRT-PCR, we confirmed that levels of cadherin-17 mRNA were significantly lower in cadherin-17(−)/FP10SC2#1 and #2 cells than in the mock transfectant (control/FP10SC2) (Figure 6(a)). Consistent with that finding, western blotting showed that cadherin-17 protein was also expressed at a lower level in cadherin-17(−)/FP10SC2#1 and #2 cells than in control/FP10SC2 cells (Figure 6(b)). By contrast, there was no difference in the proliferation rates of cadherin-17(−)/FP10SC2#1 and #2 cells and those of FP10SC2 and control/FP10SC2 cells (Figure 7(a)).

### 3.7. Cadherin-17 Knockdown Reduced Anchorage-Independent Growth and Cell Migration

**Figure 8:**
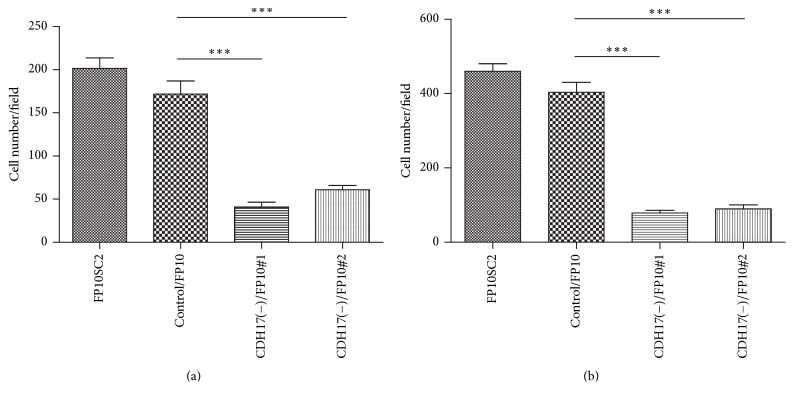
Migration of FP10SC2 cells, control/FP10SC2, CDH17(−)/FP10SC2 clone #1, and CDH17(−)/FP10SC2 clone #2. (a) Cells (4 × 10^4^ cells/well) were seeded on BMEC monolayers formed on polycarbonate porous membranes, and the numbers of the cells migrating through the were counted after 18 h. (b) Cells (4 × 10^4^ cells/well) were seeded directly on polycarbonate porous membranes, and the numbers of the cells migrating through the pores were counted after 18 h. In both panels, data are means ± SD; ^*∗∗∗*^*p* < 0.001.

High potentials for anchorage-independent cell growth and cell migration are well-known* in vitro* properties related to cancer metastasis and malignancy. We therefore used colony formation and migration assays to evaluate the effect of cadherin-17 suppression on anchorage-independent cell growth and cell migration, respectively. We found that cadherin-17(−)/FP10SC2 cells showed dramatically less anchorage-independent growth (Figure 7(b)) than control/FP10SC2 cells such that cadherin-17(−)/FP10SC2#1 and #2 cells formed almost no colonies (Figure 7(b)). Photographs of the colonies of FP10SC2 and control/FP10SC2 are shown in Figure 7(c). In addition, migration was significantly diminished in cadherin-17(−)/FP10SC2#1 and #2 cells as compared to control/FP10SC2, with (Figure 8(a)) and without a BMEC monolayer (Figure 8(b)). The mean numbers of cadherin-17(−)/FP10SC2#1 and #2 cells showing transendothelial migration were 40.8 and 61.0 respectively, while those of the FP10SC2 and control/FP10SC2 cells were 201.5 and 171.7, respectively (Figure 8(a)). In the absence of a BMEC monolayer, the mean numbers of migrated cadherin-17(−)/FP10SC2#1 and #2 cells was 78.5 and 89.5, respectively, while those of FP10SC2 and control/FP10SC2 cells were 460 and 403, respectively (Figure 8(b)).

### 3.8. Cadherin-17(−)/FP10SC2 Cells Showed Reduced Metastasis to Bone Marrow

When cadherin-17(−)/FP10SC2 cells were subcutaneously injected to BALB/c mice, the incidence of metastasis to spine was 25% for cadherin-17(−)/FP10SC2#1 cells and 47% for cadherin-17(−)/FP10SC2#2 cell (Table 2). By contrast the incidence of spinal metastasis for control/FP10SC2 cells was 85%. Moreover, the incidence of metastasis to lung was 75% for cadherin-17(−)/FP10SC2#1 cells but was 100% for control/FP10SC2 cells. Thus expression of cadherin-17 clearly enhances the metastatic activity of breast cancer cells in our model.

We further analyzed percent metastatic area per lung or spine in the histological sections using Image J software. As a result, the percent metastatic area in the lung of the mice injected by CDH17(−)/FP#1 and #2 was almost the same as that by control/FP10SC2 (approximately 60%). On the other hand, percent metastatic area in the spine of the mice injected by CDH17(−)/FP#1 and #2 was significantly decreased (12.4 and 9.7%, resp.) compared to that in the spine of the mice injected by control/FP10SC2 (42.6%). Although the incidence of metastasis of CDH17(−)/FP#1 (25%) was lower than that of CDH17(−)/FP#2 (47%), the percent metastatic area in the spine of CDH17(−)/FP#1 injected mice (12.4%) was rather higher than that of CDH17(−)/FP#2 injected mice (9.7%). As a result, we estimate that there is not so much difference in the metastatic activity of these two subclones. When the sections of metastatic tumors developed by CDH17(−)/FP#1 and #2 were stained with anti-cadherin-17 antibody, the expression of cadherin-17 was not detected.

## 4. Discussion

Cadherin-17 reportedly mediates significant increases in the adhesion and proliferation of highly metastatic human colorectal cancer cells, and cadherin-17 silencing in these cells suppresses tumor growth and liver metastasis [15]. In addition, the prognosis of gastric and ovarian cancer patients with tumors expressing cadherin-17 is significantly worse than that of patients whose tumors do not express cadherin-17 [16–18]. However, little is known about the actions of cadherin-17 in breast cancer metastasis.

We previously established 4T1E/M3 highly bone marrow metastatic breast cancer cells. The metastatic potential of 4T1E/M3 cells to lung (100%) and spine (85% to 100%) was high when intravenously injected into mice but was rather low (about 20%) when injected subcutaneously [3]. However, by passing 4T1E/M3 cells through a number of* in vitro* and* in vivo* selection steps (see Materials and Methods for details), we established FP10SC2 cells, which exhibited higher metastatic activity when subcutaneously injected into mice (Table 1). A notable difference between FP10SC2 and the parental 4T1E/M3 cells is the higher expression of cadherin-17 in FP10SC2 cells (Figures 4(a) and 4(b)). Furthermore, although suppressing cadherin-17 in FP10SC2 cells had no effect on their proliferation (Figure 7(a)), the* in vitro* anchorage-independent growth and migration activity of FP10SC2 cells were markedly decreased (Figures 7(b), 7(c), 8(a), and 8(b)), as was* in vivo* pulmonary and spinal metastasis (Table 2). Because 4T1E/M3 breast cancer cells originally have very strong metastatic activity to lung (almost 100%), it seems to be difficult to suppress the metastasis to lung strongly. As a result, the knockdown of cadherin-17 may look more effective on the suppression of spine metastasis (Table 2).

It was reported that downregulation of cadherin-17 inactivates WNT signaling and inhibits tumor growth in hepatocellular carcinoma and gastric cancer [9–11, 19]. The involvement of WNT signaling was also shown in human breast cancer cell line, MDA-MB-231 [20]. It was demonstrated that downregulation of cadherin-17 induced inactivation of NFkB signaling pathway with the reductions of downstream proteins including VEGF-C and MMP-9 and suppressed proliferation, adherence, and invasion in gastric cancer [21]. It was further shown that targeting cadherin-17 inactivated Ras/Raf/MEK/ERK signaling and inhibited cell proliferation in gastric cancer [22]. Moreover, cadherin-17 was shown to modulate *α*1*β*2 integrin signaling to induce specific focal adhesion kinase and Ras activation and led to the increase in cell adhesion and proliferation in colon cancer cells and the RGD motif in cadherin-17 was important in this process [14, 23]. Although the underling mechanism of the effect of cadherin-17 suppression in our model is still unclear, the same mechanism above demonstrated may be involved in the decrease of metastasis in the lung and spine. We consider that analysis of the potential interaction of cadherin-17 with the molecules in signaling pathways will be an important future study in our model.

In the studies on many tumors, for example, gastric, ovarian, hepatocellular, and colorectal cancer, expression of cadherin-17 in human tissues was examined and estimated as an important candidate of prognosis marker [16, 24]. Examination of cadherin 17 expression in human breast cancer tissues may be useful to detect a therapeutic target for future research.

Taken together, these findings suggest that expression of cadherin-17 promotes the metastatic activity of the highly bone marrow metastatic breast cancer cells in our model, and that cadherin-17 expression may be a useful marker of bone marrow metastasis in breast cancer.

## 5. Conclusion

In this study, we concluded that the expression of cadherin-17 promotes the metastatic activity of bone marrow metastatic breast cancer cells and that cadherin-17 may be a useful marker of bone marrow metastasis in breast cancer.

## Figures and Tables

**Table 1:** Incidences of metastasis by 4T1E/M3 and FP10SC2.

Cell	Lung	Spine
4T1E/M3	100% (6/6)	33% (2/6)
FP10SC2	100% (13/13)	85% (11/13)

*Note.* 4T1E/M3 (*n* = 6) or FP10SC2 (*n* = 13) cells (1 × 10^6^/mouse) were subcutaneously injected to mice and 25 days later tissue metastasis was evaluated in histological sections.

**Table 2:** Incidences of metastasis and percent metastatic area per tissue by control/FP10SC2, CDH17(−)/FP#1, and CDH17(−)/FP#2.

Cell	Lung	Spine
Control/FP10SC2	100% (13/13)	85% (11/13)
	60.5 ± 3.35	42.6 ± 2.88

CDH17(−)/FP#1	75% (9/12)	25% (3/12)
	58.3 ± 4.23	12.4 ± 3.57

CDH17(−)FP#2	100% (15/15)	47% (7/15)
	56.8 ± 3.68	9.7 ± 2.48

*Note*. Control/FP10SC2 (*n* = 13), CDH17(−)/FP#1 (*n* = 12), or CDH17(−)/FP#2 (*n* = 15) cells (1 × 10^6^/mouse) were subcutaneously injected to mice and 25 days later tissue metastasis was evaluated in histological sections. Percent metastatic area per lung or spine was calculated using Image J.
